# P-752. Do Antibiotics Improve Wound Necrosis Following Biodegradable Temporizing Matrix (BTM) Placement in Patients with Xylazine-associated Wounds?

**DOI:** 10.1093/ofid/ofae631.948

**Published:** 2025-01-29

**Authors:** Sara K Schultz, Lisa Rae, Oumaima Bougazzoul

**Affiliations:** Temple University Hospital, Philadelphia, Pennsylvania; Temple University Hospital, Philadelphia, Pennsylvania; Temple University Hospital, Philadelphia, Pennsylvania

## Abstract

**Background:**

Xylazine-associated wounds are a unique wound typology among patients with injection behavior in geographic areas where xylazine has infiltrated the fentanyl drug supply. The use of the dermal substitute biodegradable temporizing matrix (BTM) is being evaluated to promote wound stabilization in the presence of severe soft tissue necrosis, a well-known characteristic of xylazine wounds. The role of antibiotics in the treatment of BTM placement for xylazine wounds is unclear.

**Methods:**

All patients with xylazine-associated wounds who underwent BTM placement at Temple University Hospital since August 2023 were identified. The electronic medical record was used to collect data including demographics, antibiotic course, microbiologic data, discharge disposition, and appearance of the wound. The primary outcome was evidence of less necrotic tissue on a stable wound within 60 days.

**Results:**

A total of 37 patients were identified. 60% of patients had an infectious disease consult. 89% of patients had clinical or radiographic evidence of osteomyelitis underlying their xylazine wound. 25 patients (68%) received antibiotics during their admission for BTM. 15 (41%) of patients received at least 4 weeks of antibiotics. 12 patients (33%) did not receive any antibiotics during their admission. 25% of patients received antibiotics for an indication unrelated to their xylazine wound. Treatment regimens varied and included both oral and intravenous antibiotics. 49% of patients lacked stable housing at the time of discharge. 13 (35%) patients had evidence of less necrotic tissue on a stable wound at 60 days. Of those 13 patients, 9 received antibiotics and 4 received none. Of the patients who received at least 4 weeks of antibiotics, only 11% had evidence of less necrotic tissue at 60 days. Of the patients who received no antibiotics, 8% had evidence of less necrotic tissue at 60 days.

**Conclusion:**

Severe tissue necrosis related to injection of xylaxine requires new treatment paradigms. Our small study questions the utility of prolonged antibiotic treatment courses for patients with xylazine wounds and BTM placement, including in the setting of osteomyelitis, as we did not see high treatment success in these patients.

**Disclosures:**

**Sara K. Schultz, MD FACP FIDSA**, AbbVie: Advisor/Consultant

